# Two new species of *Liljeborgia* Spence Bate, 1863 (Crustacea, Amphipoda, Liljeborgiidae) from the Clarion-Clipperton Zone, Pacific Ocean

**DOI:** 10.3897/zookeys.1274.140236

**Published:** 2026-03-24

**Authors:** Roxana Timm, Eva C. D. Stewart, Anne-Nina Lörz, Tammy Horton

**Affiliations:** 1 Institute of Marine Ecosystem and Fishery Science (IMF), Center for Earth System Research and Sustainability (CEN), University of Hamburg, Große Elbstraße 133, 22767 Hamburg, Germany National Oceanography Centre Southampton United Kingdom; 2 School of Ocean and Earth Sciences, University of Southampton, Southampton, SO14 3ZH, UK University of Hamburg Hamburg Germany; 3 Life Sciences Department, Natural History Museum, London, Cromwell Road, South Kensington, SW7 5BD, UK University of Southampton Southampton United Kingdom; 4 Marine Research at Senckenberg am Meer, Südstrand 40, 26382 Wilhelmshaven, Germany Life Sciences Department, Natural History Museum London United Kingdom; 5 National Oceanography Centre, Southampton, SO14 3ZH, UK Marine Research at Senckenberg am Meer Wilhelmshaven Germany

**Keywords:** Abyssal, amphipods, deep-sea, liljeborgiids, taxonomy

## Abstract

Two new species of *Liljeborgia* are described from the Clarion-Clipperton Zone, central-east Pacific Ocean. *Liljeborgia scylla***sp. nov**. is described from a single immature male specimen collected at 4223 m depth. This species is characterised by a tooth-like protrusion on the second gnathopod, a very narrow basis of pereopods 5–7, and a shallow cleft in the telson. *Liljeborgia sibylline***sp. nov**. was collected at depths ranging from 4107 to 4287 m and is described from six specimens. Despite similarities in morphology to *Liljeborgia scylla***sp. nov**., *Liljeborgia sibylline***sp. nov**. can be distinguished by characters of the gnathopods, telson, and epimeron 3. Molecular barcode data are also provided for both species, offering additional insights into their genetic distinctiveness.

## Introduction

*Liljeborgia* Spence Bate, 1863 is a species-rich genus with 75 described species, displaying a circumglobal distribution and a broad bathymetric range (0–6000 m) (Barnard and Karaman 1991). The genus belongs to the family Liljeborgiidae Stebbing, 1899 which comprises 121 primarily benthic species, characterised as micro-predators (Brix et al. 2018; Horton et al. 2024). The family is divided into two subfamilies, the Idunellinae d’Udekem d’Acoz, 2010, which includes the genera *Idunella* G.O. Sars, 1894 and *Sextonia* Chevreux, 1920, and the Liljeborgiinae Stebbing, 1899, a monogeneric subfamily containing the genus *Liljeborgia*. d’Udekem d’Acoz (2010a) further divided the genus *Liljeborgia* into three groups. The subgenus *Liljeborgia* Spence Bate, 1863 (type species: *Gammarus pallidus* Spence Bate, 1857) contains 29 species (33 incl. undescribed), while the subgenus *Lilljeborgiella* Schellenberg, 1931 (type species: *Lilljeborgiella longicornis* Schellenberg, 1931) contains 37 species (39 incl. undescribed). The third group includes some poorly known Northwestern Pacific species and a South African species and was not formally designated as a subgenus (d’Udekem d’Acoz 2010a). Both subgenera are cosmopolitan and found at a wide bathymetric range, but Liljeborgia (Lilljeborgiella) is more diverse in cold and deep waters (d’Udekem d’Acoz and Hendrycks 2011).

There are thirteen recorded species of *Liljeborgia* that are currently known to live at depths of more than 3000 m. In the Southern Ocean, four deep water species are described, including *L. abyssotypica* d’Udekem d’Acoz, 2008 from the Tasman Basin at 4304 m; *L. bythiana* d’Udekem d’Acoz, 2008 from the western Weddell Sea at 3050 m; *L. homospora* d’Udekem d’Acoz, 2008 from the eastern and western Weddell Sea at 4385–4392 m; and *L. permacra* d’Udekem d’Acoz, 2008 found in the South of the Drake Passage from 3622–3643 m (d’Udekem d’Acoz 2008). The deep cold waters of the Arctic are home to *L. polosi* Barnard & Karaman, 1991, which has been found in the Canadian Basin at 2710–3550 m (Kamenskaya 1980 as *L. dubia*; Barnard and Karaman 1991; d’Udekem d’Acoz and Vader 2009). In the Indian Ocean there are three abyssal species known: *L. gloriosae* Ledoyer, 1986 at 3700 m; *L. mojada* J. L. Barnard, 1961 at 3716–5090 m; and *L. mozambica*Ledoyer 1986 at 3370 m.

The species *L. charybdis* d’Udekem d’Acoz & Vader, 2009 was described from the Norwegian Sea and Svalbard in the North Atlantic at 740–3678 m (d’Udekem d’Acoz and Vader 2009; d’Udekem d’Acoz 2010a). In the equatorial eastern Atlantic, the species *L. famelicosa* d’Udekem d’Acoz & Hendrycks 2011, was found at 5142 m. In the southeast Atlantic, the species *L. zarica* J.L. Barnard, 1961 is described from 4893 m (Barnard 1961, 1962).

To date, only two abyssal *Liljeborgia* species have been described from the Pacific Ocean. The only known record of *L. holthuisi*d’Udekem d’Acoz 2010b was from the tropical southeast Pacific at 5825–5841 m; while the species *L. caeca* Birstein & Vinogradova, 1960 was described from the northwest Pacific at 6156–6207 m.

In this study, we add to the knowledge of abyssal *Liljeborgia* species from the Pacific Ocean by describing two new species of the genus *Liljeborgia* from the Clarion-Clipperton Zone (CCZ).

## Materials and methods

### Specimen preparation

The material for the present study was sampled in the central-east Pacific, specifically in the easternmost sector of the Clarion-Clipperton Zone (CCZ). The material studied was collected using an USNEL box corer (BC) and epibenthic sledge (EBS) during five expeditions to three different exploration contract areas (henceforth, contract areas) in the CCZ: the OMS, the NORI-D, and the BGR contract areas. For details of gear deployment and sample processing see Jażdżewska and Horton (2026).

Individuals were initially examined using either a Leica M125 or a Nikon SMZ800 stereomicroscope. The habitus of *Liljeborgia scylla* sp. nov. and *Liljeborgia sibylline* sp. nov. are presented as photographs obtained with a confocal laser scanning microscope (CLSM). The holotypes were stained in Congo red and acid fuchsin, temporarily mounted onto slides with glycerin and examined with a Leica TCS SPV equipped with a Leica DM5000 B upright microscope and three visible-light lasers (DPSS 10 mW 561 nm; HeNe 10 mW 633 nm; Ar 100 mW 458, 476, 488 and 514 nm), combined with the software LAS AF 2.2.1 (Leica Application Suite, Advanced Fluorescence). A series of photographic stacks were obtained, collecting overlapping optical sections throughout the whole preparation (Michels and Büntzow 2010; Kamanli et al. 2017).

The holotypes were dissected and mounted on permanent slides using polyvinyl-lactophenol containing lignin pink. All slides were examined using either a Leica M125 microscope or a Leitz Diaplan Type 020-437.035, via a camera lucida.

The pencil drawings were scanned and digitally inked with Adobe Illustrator CS6 and CorelDRAW Graphics Suite 2023 following techniques in Coleman (2003, 2009). Different drawing tablets were used for inking, mainly Wacom Intuos CTH-480 and Wacom DTF-720. Figures were edited in CorelDRAW and ClipStudio Paint Pro version 1.10.5. In the descriptions and figures the following abbreviations were used: **A1, A2** = antenna 1, 2; **c1–c4** = coxa 1–4; **G1, G2** = gnathopod 1, 2; **LL** = lower lip; **Md** = mandible; **Mx1, Mx2** = maxilla 1, 2; **Mxp** = maxilliped; **P3–P7** = pereopod 3–7; **T** = telson; **U1–U3** = uropod 1–3; **UL** = upper lip; **l** = left; **r** = right. The terminology for the setae and spines follows the work of Garm and Watling (2013). A “tooth” describes a pointed ectodermic structure. To simplify species’ descriptions, the expression ‘N×’ replaces ‘N times longer than’ and ‘length N × width’ replaces ‘N times as long as wide’.

Type material is deposited in the Natural History Museum, London (**NHMUK**) and the Senckenberg Museum (Frankfurt, Germany) (**SMF**). Additional material is kept in the Deutsches Zentrum für Marine Biodiversitätsforschung (**DZMB**) in Wilhelmshaven and the Discovery Collections at the National Oceanography Centre, Southampton (**DISCOLL**).

### DNA extraction, amplification, and sequencing

Specimens collected from ABYSSLINE-2 and MANGAN cruises were extracted and sequenced as described in Jażdżewska et al. (2025). Specimens collected from the NORI-D area were processed as follows.

DNA was extracted from a pair of pleopods using QuickExtract^TM^ DNA extraction solution (Lucigen), following manufacturer guidelines, and adapted for a digestion time of 45 minutes. Regions of two mitochondrial [16S rRNA (16S) and cytochrome oxidase subunit I (COI)] and three nuclear [28S rRNA (28S), and early-stage histone 3 (H3)] genetic markers were amplified with published primer sets (Folmer et al. 1994; Corrigan et al. 2014; Lörz et al. 2018). The PCR mix for each reaction contained 10.5 µl of Red Taq DNA Polymerase 1.1X MasterMix (VWR), 0.5 µl of each primer (10 µM), and 1 µl of DNA template. Details of primers and PCR conditions can be found in Stewart et al. (2024).

The primers used for sequencing were the same as those for amplifications. PCR products were purified using a Millipore Multiscreen 96-well PCR Purification System and sequenced using an ABI 3730XL DNA Analyzer (Applied Biosystems) at The Natural History Museum Sequencing Facilities. For each gene fragment contigs were assembled by aligning both forward and reverse sequences, chromatograms were visually inspected, and ambiguous base calls were corrected manually, using Geneious 7.0.6 (Kearse et al. 2012).

The relevant voucher information, taxonomic classifications, and sequences are deposited in the data set “DS-AMPHICCZ” in the Barcode of Life Data System (BOLD) (https://doi.org/10.5883/DS-AMPHICCZ) (www.boldsystems.org) (Ratnasingham and Hebert 2007).

## Results

### Systematics

**Order AMPHIPODA Latreille, 1816**

**Suborder AMPHILOCHIDEA Boeck, 1871**

**Family LILJEBORGIIDAE Stebbing, 1899**

**Genus *Liljeborgia* Spence Bate, 1863**

**Subgenus *Lilljeborgiella* Schellenberg, 1931**

#### Liljeborgia (Lilljeborgiella) scylla sp. nov.

Taxon classificationAnimaliaAmphipodaLiljeborgiidae

6541E37B-EE6C-58B1-ABA2-D88ACB9CAAA7

https://zoobank.org/0D591FA7-A19C-41B0-BB26-533D896C50C1

**Figure 1.**
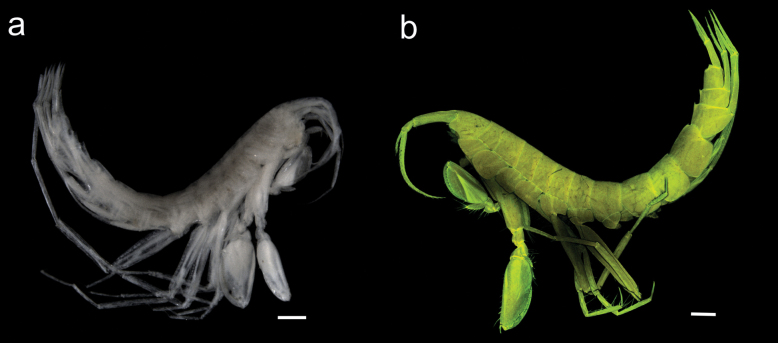
Habitus of *Liljeborgia scylla* sp. nov. **A** light microscopy image of holotype SMF 62821, **B** maximum intensity projection of CLSM image stacks of holotype SMF 62821. Scale bar: 500 µm.

**Figure 2.**
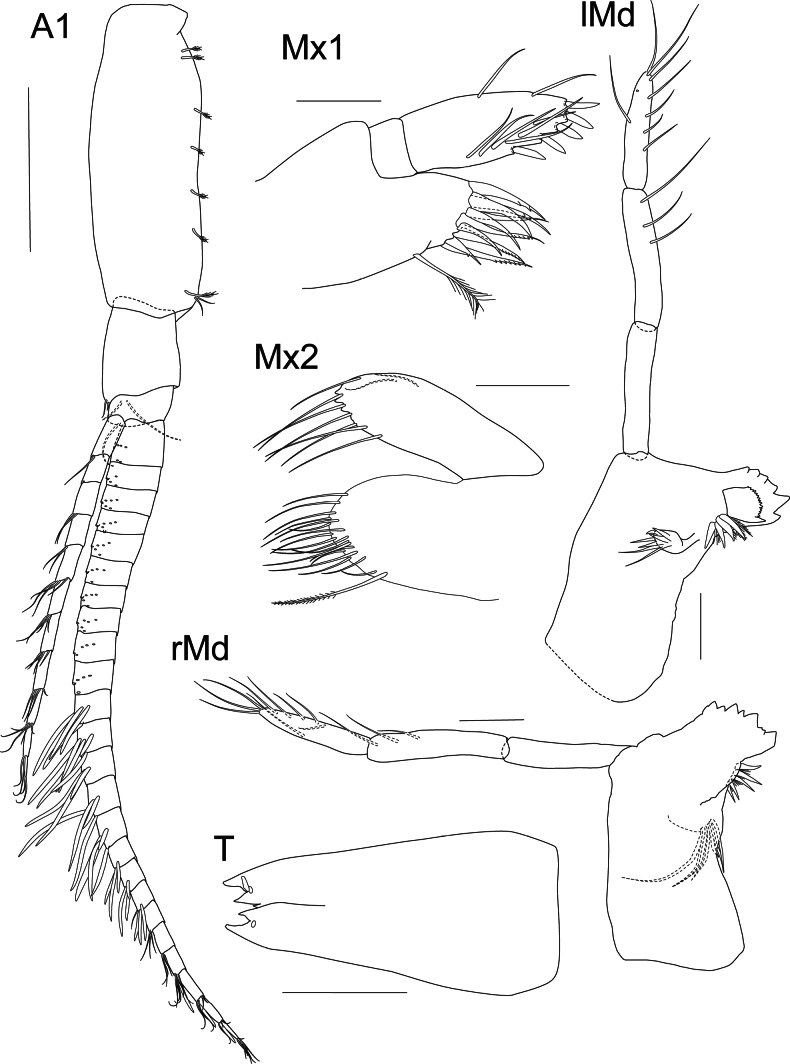
*Liljeborgia scylla* sp. nov., holotype: SMF 62821, male, 7 mm, Clarion-Clipperton Zone. Scale bars: 0.5 mm (A1); 0.1 mm (Mx1, Mx2, lMd, rMd, T).

**Figure 3.**
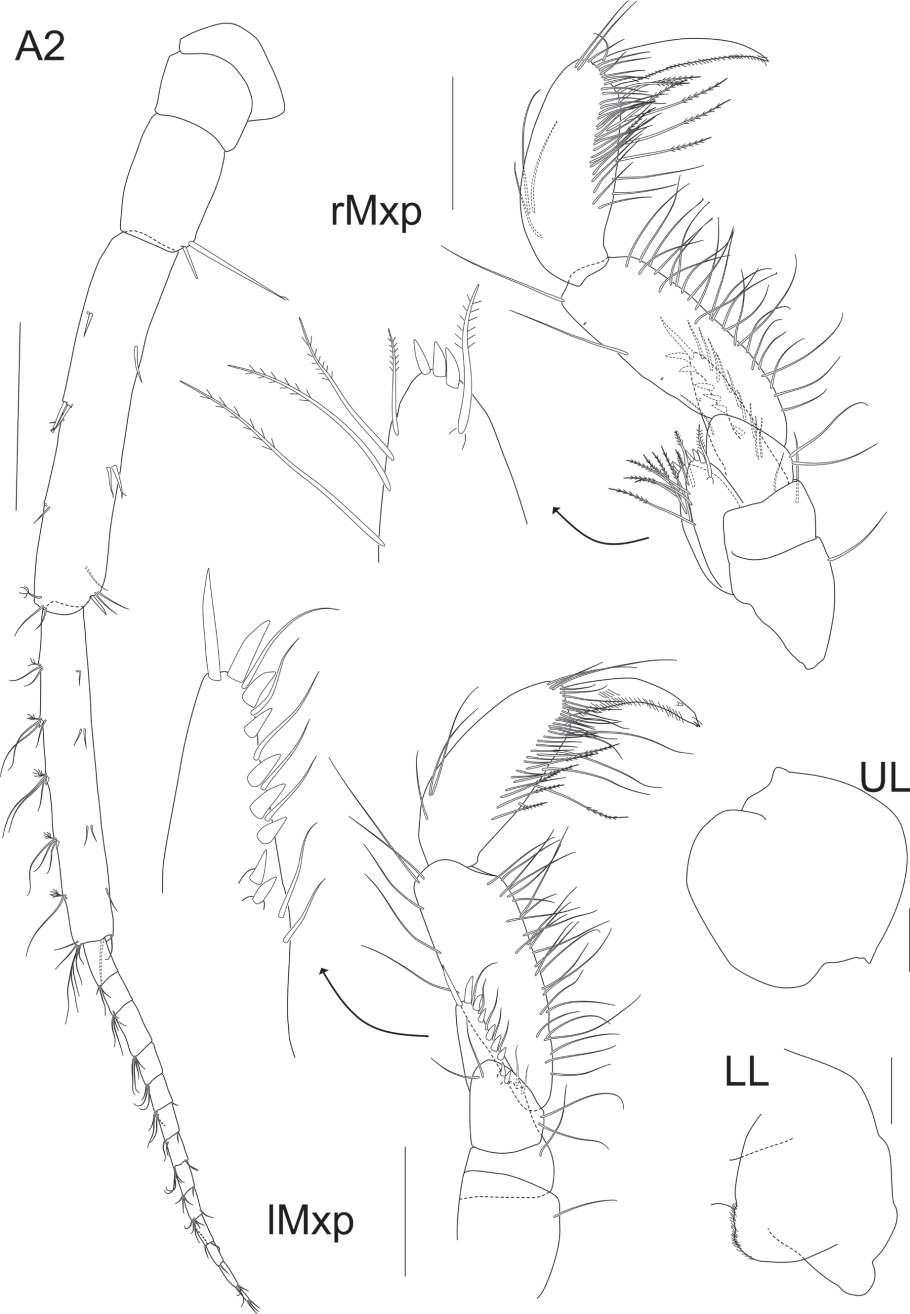
*Liljeborgia scylla* sp. nov., holotype: SMF 62821, male, 7 mm, Clarion-Clipperton Zone. Scale bars: 0.5 mm (A2); 0.25 mm (rMxp, lMxp); 0.1 mm (UL, LL).

**Figure 4.**
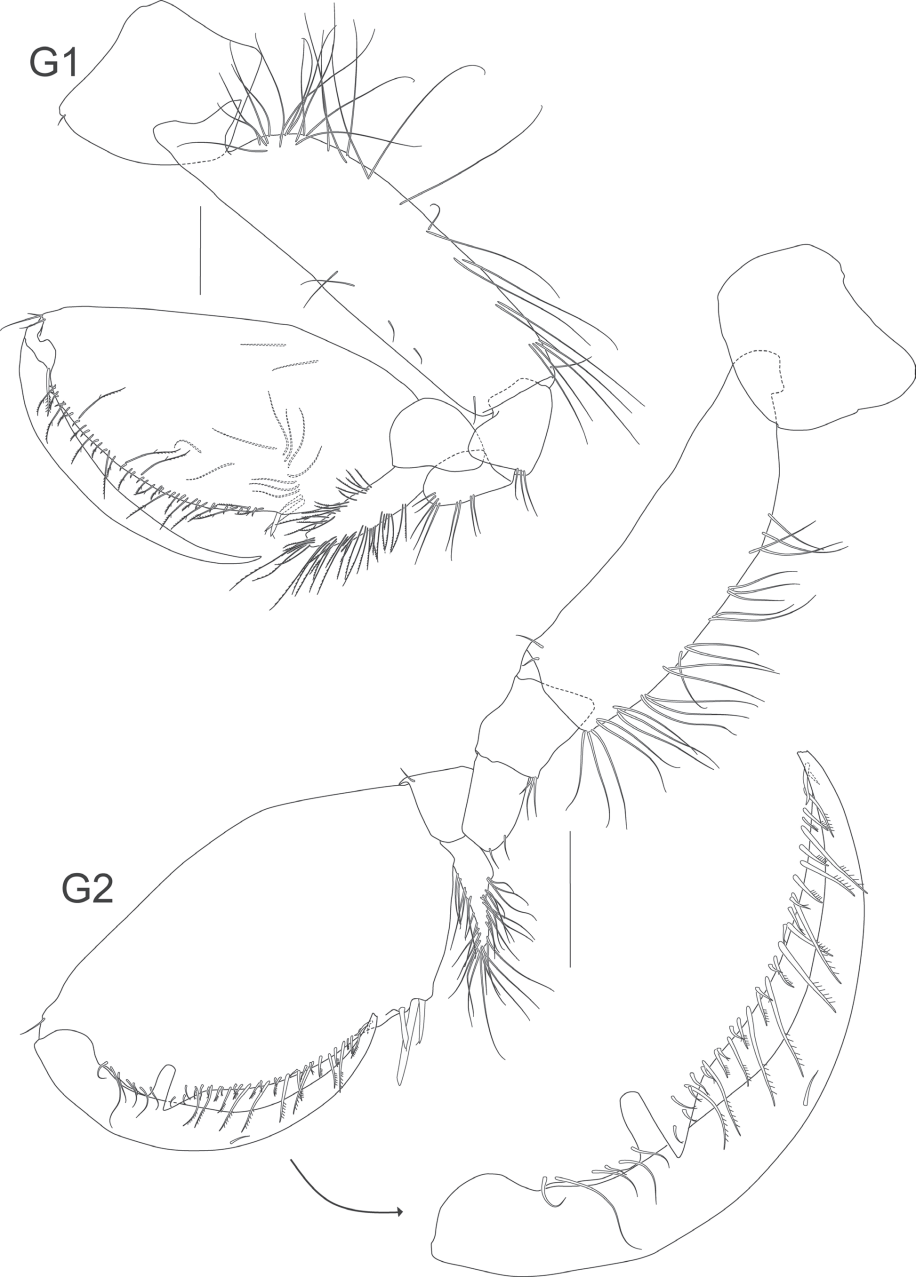
*Liljeborgia scylla* sp. nov., holotype: SMF 62821, male, 7 mm, Clarion-Clipperton Zone. Scale bars: 0.25 mm (G1); 0.5 mm (G2).

**Figure 5.**
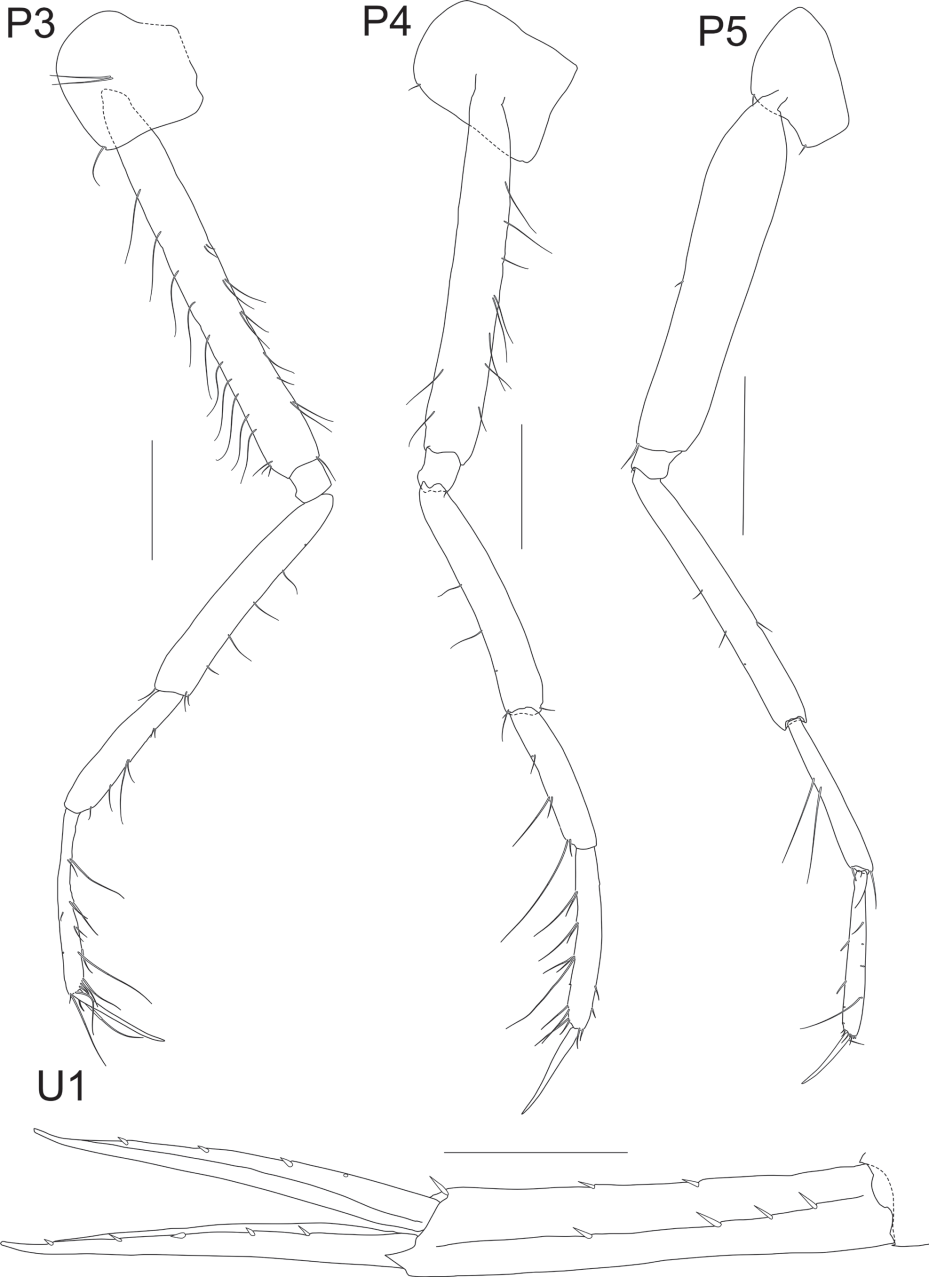
*Liljeborgia scylla* sp. nov., holotype: SMF 62821, male, 7 mm, Clarion-Clipperton Zone. Scale bars: 0.5 mm (P3, P4, U1); 0.2 mm (P5).

**Figure 6.**
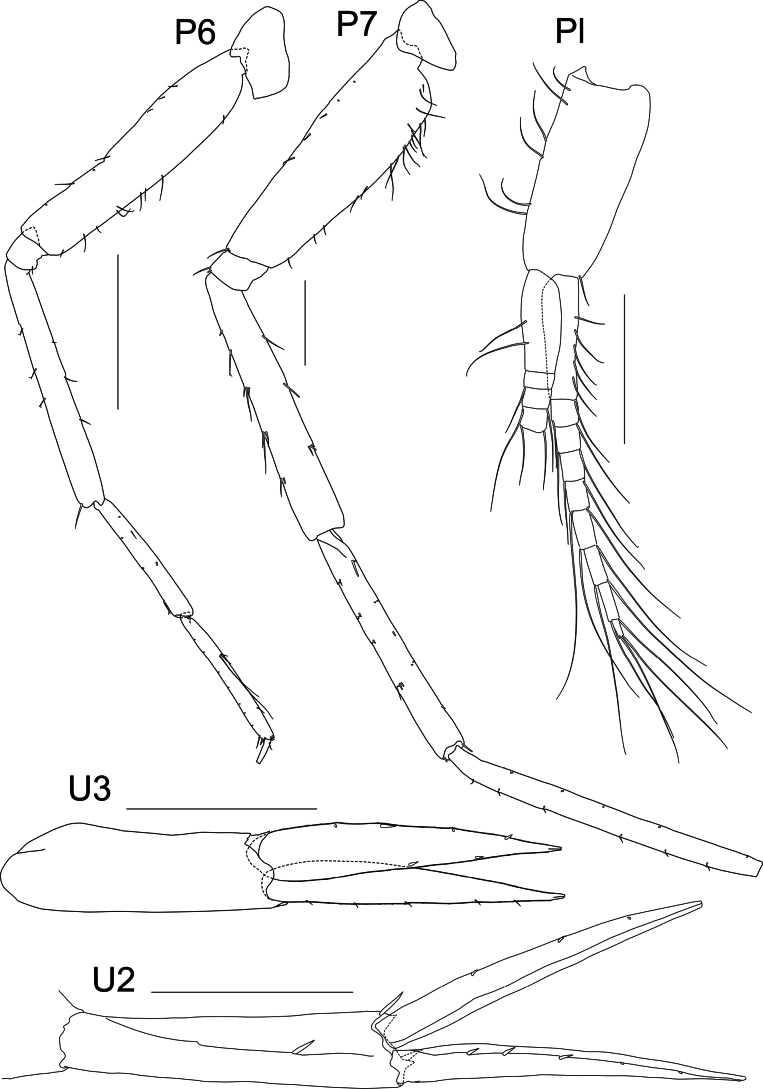
*Liljeborgia scylla* sp. nov., holotype: SMF 62821, male, 7 mm, Clarion-Clipperton Zone. Scale bars: 0.5 mm (P6, P7, Pl, U3, U2).

Figs 1, 2, 3, 4, 5, 6

##### Type material.

***Holotype***: Pacific • Immature male, 7 mm, carcass and two slides; Clarion-Clipperton Zone; 12.045°N, 117.424°W, depth 4223 m; 01/03/2015; OMS contract area, “RV Thompson”, Cruise ABYSSLINE-2, Station AB2-EB-06; epibenthic sledge; SMF 62821; COI (PQ734561).

##### Type locality.

Abyssal Pacific Ocean, Clarion-Clipperton Zone, 12.045°N, 117.424°W, depth 4223 m.

##### Diagnosis.

Maxilliped palp article one with dorsal distal setae, article three anterior margin with setae in groups or pairs. Palmar margin of gnathopod 2 of young males with prominent notch followed by triangular protrusion. Pereopods 3 and 4 propodus posterior border with long setae. Pereopods 5–7 bases very narrow, lacking crenulations on the posterior margin. Pleosomites 2 and 3 posterodorsal area produced into a tooth; epimera 1–3 rounded posterodistally. Uropod 1 peduncle dorsomedial margin with several spines. Telson shallowly cleft, 30%, inter-teeth spine probably barely reaching tip of medial tooth. Eyes absent.

##### Description.

Based on holotype immature male, 7 mm, SMF 62821.

***Head*** (Figs 1, 2, 3): ***Rostrum*** small, reaching 25% of article 1 of peduncle of A1. ***Eyes*** absent, colourless in alcohol. ***Antenna 1*** primary flagellum with 25 articles, with aesthetascs; accessory flagellum with 11 articles. ***Antenna 2*** article four of peduncle with setae and with dorsomedial and ventrolateral spines; article five with dorsomedial setae; flagellum with 13 articles. ***Lower lip*** covered in fine setules. ***Upper lip*** rounded, not setose.

***Mouthparts*** (Figs 2, 3): ***Mandible*** right anterior margin with six triangular teeth, one of them large; left anterior margin with seven triangular teeth. Incisor process with five raker spines. Article one of palp length ~ 6.04 × width (ratio length article one / article two ~ 0.93); article two with setae along the distal margin, length ~ 5.44 × width; article three with nine setae along the margin and at the distal part of the margin, length 4.5 × width, 0.82 × article two. ***Maxilla 1*** palp second article with two long setae on lateral margin, five slender spines on apical and medial margins and six slender facial setae; outer plate with ten robust spines, of which six are strongly denticulate; inner plate with a single seta on tip. ***Maxilla 2*** setose, outer plate with two setae on anterolateral margin. ***Maxilliped*** inner plate with three anterior spines and five marginal and submarginal plumose setae; outer plate with nine evenly-spaced short, stout spines on medial border, interspersed with seven medioventral slender setae; palp article one with two distal and dorsal setae; article two with dorsomedial setae, with non-distal setae on outer margin; palp article three 0.8 × article two with two transverse rows of dorsomedial setae, one consisting of seven strong, plumose setae; dactylus 0.8 × article three, with anterior and posterior margins distinctly curved, posterior margin covered with fine setules, anterior margin with setae in groups or pairs.

***Pereon*** (Figs 4, 5, 6): ***Gnathopod 1*** coxa broadly quadrate with anteroventral notch that is associated with a seta; merus with three groups of two to five setae; carpus with confluent groups of setae; carpus prolongation reaching just beyond palmar defining spine group; propodus twice as long as wide; palm border very slightly convex; with small, hooked spines and slender, long setae; dactylus without teeth. ***Gnathopod 2*** coxa broadly rectangular; merus and carpus with groups of setae; merus with two solitary setae; carpus with confluent groups of setae; carpus prolongation almost reaching palmar defining spine group; propodus length 1.79 × width; palm border convex, with small hooked spines and slender, long setae; palmar margin with a prominent notch followed by triangular protrusion that appears to accept the dactylus; dactylus without teeth; G2 larger than G1; ratio length of propodus of Gn2/length of propodus of Gn1: 1.4. ***Pereopod 3*** coxa with one seta at anteroventral border; merus 1.59 × carpus and 1.41 × propodus; carpus length 5 × width; propodus length 8.78 × width; dactylus slender, slightly curved, medium length, 0.63 × carpus and 0.56 × propodus; propodus with long setae along posterior margin small spine distally. ***Pereopod 4*** coxa with anteroventral notch with one seta; merus 1.76 × carpus and 1.52 × propodus; carpus length 4.5 × width; propodus length 6.64 × width; dactylus slender, slightly curved, medium length, 0.67 × carpus and 0.58 × propodus; propodus with long setae along posterior margin; anterior border with one small seta. ***Pereopod 5*** coxa with very small posterior notch, basis very narrow (length 4.97 × width), posterior border completely smooth without teeth or crenulations; carpus 0.55 × merus; carpus and propodus 1.08 × merus; propodus with long setae along posterior margin; anterior border with one small seta. ***Pereopod 6*** coxa with small posterior notch; basis very narrow (length 5.2 × width), posterior border completely smooth without teeth or crenulations; carpus 0.96 × merus; propodus with seven broken off spines or setae at the anterior border, three groups of setae with one to three setae on the posterior border and six setae at the tip; dactylus with short seta. ***Pereopod 7*** coxa without posterior tooth; basis very narrow (length 3.8 × width), posterior border completely smooth without teeth or crenulations; ischium with two small setae; carpus 0.97 × merus; propodus of pereopod 7 double the length of propodus of pereopod 6, propodus with five spines on the posterior border and six groups of setae consisting of one to two setae on the anterior border.

***Pleosome*** (Fig. 1): ***Pleonite 1*** posterodorsal area with no tooth; epimeron 1 rounded, with posterior border slightly convex. ***Pleonite 2*** posterodorsal area produced into a tooth; epimeron 2 rounded, with posterior border slightly convex. ***Pleonite 3*** posterodorsal area produced into a tooth; epimeron 3 rounded, with posterior border almost straight (very weakly convex).

***Urosome*** (Figs 2, 5, 6): ***Urosomite 1*** with a distinct middorsal tooth; peduncle of uropod 1 with two dorsolateral spines and one spine distally; four dorsomedial spines; outer ramus lateral margin with five spines and no medial spines, one small spine at the tip of ramus; inner ramus lateral margin without spines and medial margin with four spines. ***Urosomite 2*** with a distinct middorsal tooth; peduncle of uropod 2 with two dorsomedial spines; outer ramus lateral margin with three small spines; inner ramus medial margin with five spines. ***Urosomite 3*** lacking middorsal tooth; outer ramus medial margin without spines, lateral margin with five setae; inner ramus with four spines on lateral margin and two spines on medial margin; lateral and medial margins of both rami microsetose. ***Telson*** cleft to 30% of its length; outer tooth of each lobe overreaching medial tooth; inter-teeth spine broken off or damaged at both sides but probably barely reaching tip of medial tooth.

##### Etymology.

The name *scylla* is a reference to the *Liljeborgia* species described by Cedric d’Udekem d’Acoz and Wim Vader: *Liljeborgia charybdis* d’Udekem d’Acoz & Vader, 2009. There is a well-known idiom - to choose between Scylla and Charybdis which means “to choose the lesser of two evils” and we think that the genus *Liljeborgia* would be incomplete with only a “*Charybdis*” and no “*Scylla*”. Scylla, like Charybdis, is a sea monster from Greek mythology that lives by a rock in the strait between Sicily and Italy.

##### Remarks.

The new species belongs to the subgenus *Lilljeborgiella* (d’Udekem d’Acoz 2010a): several spines are present on the dorsomedial margin of the peduncle of uropod 1; some or all of the setae on the anterior margin of article three of the maxilliped palp form groups or pairs, and the palp of article one possesses distal outer setae; the setae on the posterior border of the propodus of pereopods 3 and 4 are rather long and the basis of the pereopods 5–7 is not broad.

*Liljeborgia scylla* sp. nov. is morphologically very similar to the abyssal amphipod species *L. holthuisi* but can be clearly distinguished from it (Table 1). The most significant difference between the two species is the stoutness of the basis of pereopod 7. The basis is significantly narrower in *L. scylla* than in *L. holthuisi*: ~ 3.8 × as long as wide in *L. scylla* sp. nov. and 2.58 × as long as wide in *L. holthuisi*. The new species has a middorsal tooth on pleonites 1–3, but in *L. holthuisi*, these teeth are followed by a rounded edge. The urosomite 1 has a distinct middorsal tooth in *L. scylla* sp. nov. and no carina, while the urosomite 1 has a middorsal carina with two very small teeth on each side in *L. holthuisi*. The gnathopod 2 of *L. scylla* sp. nov. has a distinctive deep notch on the palmar margin. *Liljeborgia holthuisi* also has a similar but smaller protrusion in the same position as in *L. scylla* sp. nov. The original description of *L. holthuisi* is based on a single male specimen of 10.5 mm length. By examining more individuals of these two species, we may reveal whether the palmar notch is a sexually dimorphic character and whether there is ontogenetic variation. *Liljeborgia scylla* sp. nov. and *L. holthuisi* also share their occurrence in the abyssal waters of the Pacific Ocean. *Liljeborgia holthuisi* was collected at depths of 5825–5841 m in the Peru-Chile Trench (d’Udekem d’Acoz 2010b). The new species was collected ~ 4500 km away in the Clarion-Clipperton Zone at a depth of 4137 m.

**Table 1.** Comparison of the new species *L. scylla* sp. nov, *L. sibylline* sp. nov, *L. holthuisi* (character states extracted from d’Udekem d’Acoz 2010b), and *L. cota* (character states extracted from J.L. Barnard 1962).

**Species/Character**	***L. scylla* sp. nov**.	***L. sibylline* sp. nov**.	***L. holthuisi* d’Udekem d’Acoz, 2010b**	***L. cota* J.L. Barnard, 1962**
**G2: teeth on dactylus**	Not dentate	Not dentate	Not dentate	Dentate
**G2: protrusion at the palm border of males**	Large triangular protrusion	No protrusion	Very small protrusion	Notch and protrusion in young males
**P3: merus length to carpus length**	1.59×	1.44×	1.56×	1.8×
**P3: merus length to propodus length**	1.41×	1.06×	1.32×	1.2×
**P7: basis width to length**	3.8×	4.4×	2.58×	1.94×
**Pleonites: posterodorsal tooth**	Pleonites 1–3	Pleonites 1–3	Pleonites 1–3 tooth followed by a rounded edge	Variable on pleonites 1–3, largest on pleonite 2 (male) (Sexually dimorphic?)
**Epimeron 3**	Without posteroventral tooth	Rounded with minute tooth in the corner	Without posteroventral tooth	With small posteroventral tooth
**Urosome**	Urosomites 1–2 with distinct middorsal teeth	Urosomites 1–2 with distinct middorsal teeth	Urosomites 1–2 middorsal carina with two very small teeth on each	Variable from very strongly developed to nearly obsolete
**Telson: cleft %**	30	18	17	34

The species *L. cota* J.L. Barnard, 1962 is also similar in having a shallow cleft of the telson (34%). The gnathopod 2 in *L. cota* also has a very similar protrusion and notch that is more prominent in juvenile males, while older males have a smooth palm. Additionally, the gnathopod 2 of *L. cota* has a dentate dactylus, compared to the smooth dactylus of *L. scylla* sp. nov. While both species inhabit the Northeast Pacific Ocean, their depth distributions differ, with *L. cota* found at shallower depths (366–2000 m) in South California (Barnard 1962, 1967, 1971). These two species can also be differentiated by the posteroventral corner of pleonites 1–3, which is rounded in the new species, whereas *L. cota* bears a tooth. The bases of pereopods 5–7 are also narrower in *L. scylla* sp. nov.

There are two other species that have similar protrusion-like structures on the gnathopod 2 of males. *Liljeborgia quadridentata* Schellenberg, 1931 and *L. quinquedentata* Schellenberg, 1931; both species have multiple tooth-like protrusions on the palm. It has also been observed that *L. quinquedentata* has larger teeth in juveniles than in adults (d’Udekem d’Acoz 2008).

##### Distribution.

Only known from the Clarion-Clipperton Zone, at 4223 m depth.

##### Molecular data.

Sequence data for the holotype of *Liljeborgia scylla* sp. nov. is deposited in GenBank under accession number PQ734561. The species has also received a Barcode Index Number from Barcode of Life Data Systems: BOLD:AEB5438 (https://doi.org/10.5883/BOLD:AEB5438).

#### Liljeborgia sibylline sp. nov.

Taxon classificationAnimaliaAmphipodaLiljeborgiidae

3C4A4DC8-CCF4-5762-B51B-9770B44DAD91

https://zoobank.org/3B2638D8-3EB0-446A-A785-14215B3D7856

**Figure 7.**
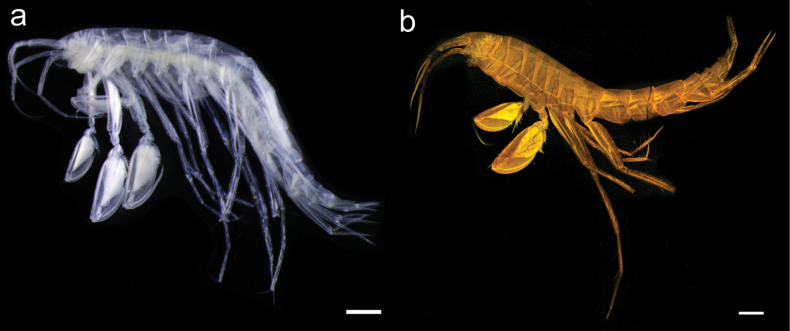
Habitus of *Liljeborgia sibylline* sp. nov. **a** paratype female, 11.44 mm, NHMUK 2025.40, Photographed on board after collection **b** holotype, immature female, 6 mm. SMF 62821, Maximum intensity projection of CLSM image stacks. Scale bars: 1 mm (**a**); 500 µm (**b**).

**Figure 8.**
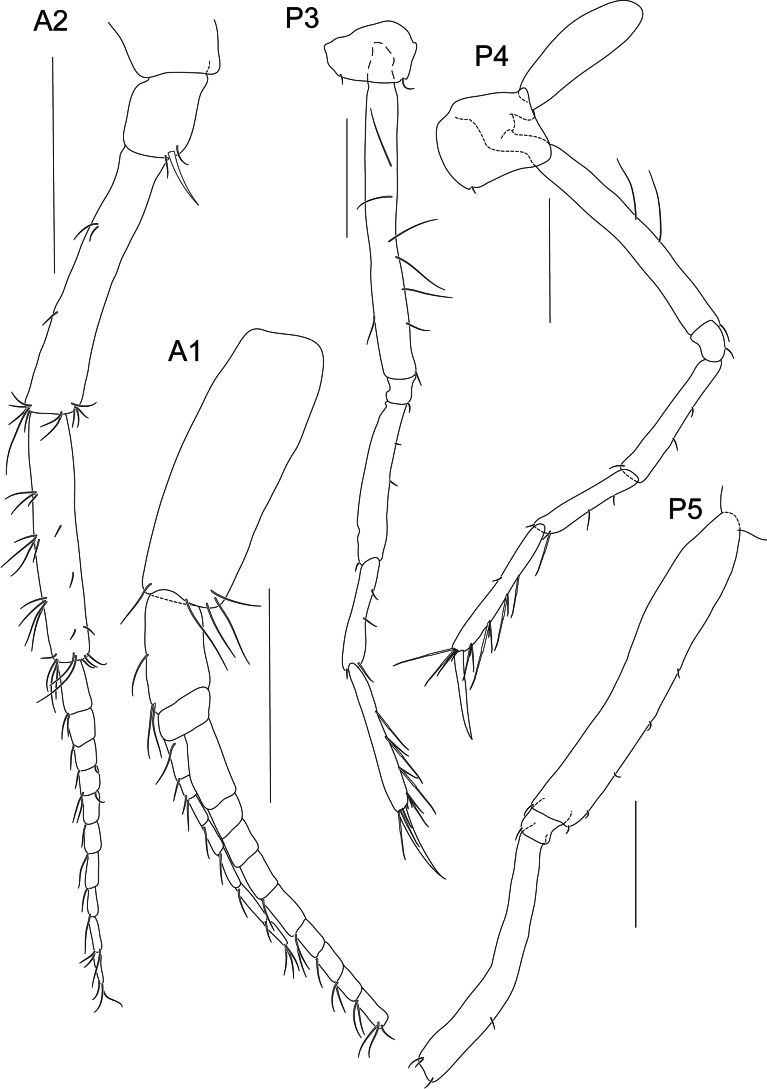
*Liljeborgia sibylline* sp. nov., holotype: SMF 62822, immature female, 6 mm, Clarion-Clipperton Zone. Scale bars: 0.5 mm (A2, A1, P3, P4, P5).

**Figure 9.**
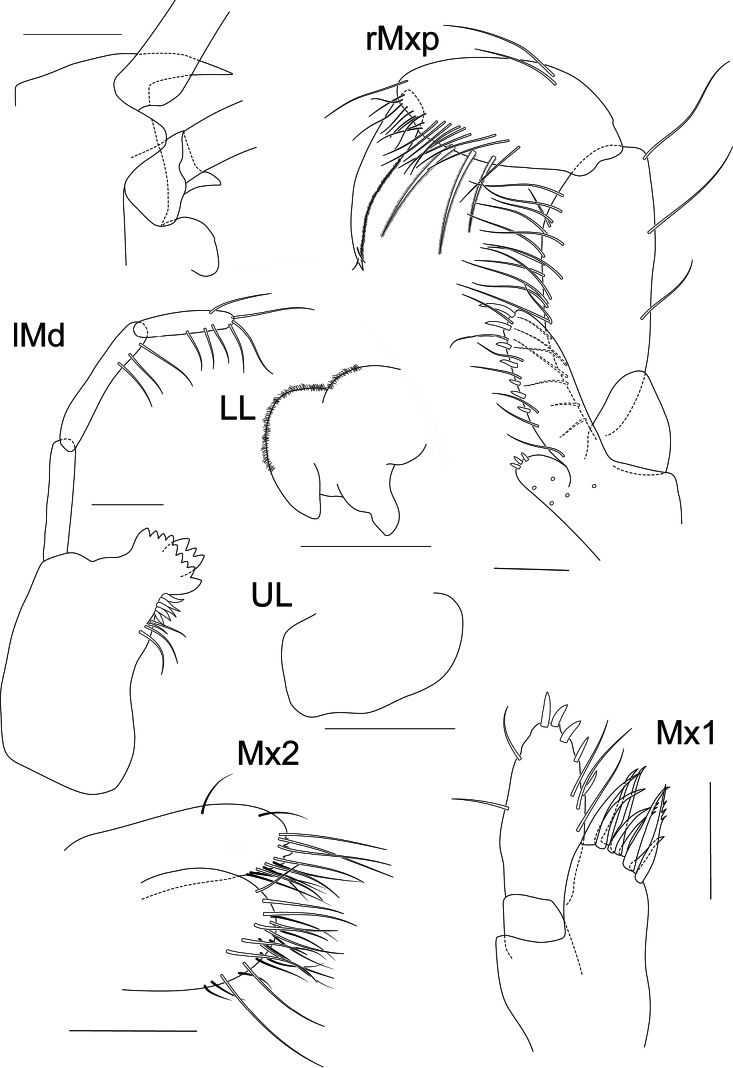
*Liljeborgia sibylline* sp. nov., holotype: SMF 62822, immature female, 6 mm, Clarion-Clipperton Zone. Scale bars: 0.1 mm (Lower lip, Mxp, Left Md, Upper lip, Mx2, Mx1); paratype NHM 2025.40, Scale bars: 0.5 mm (head, rostrum and epistome).

**Figure 10.**
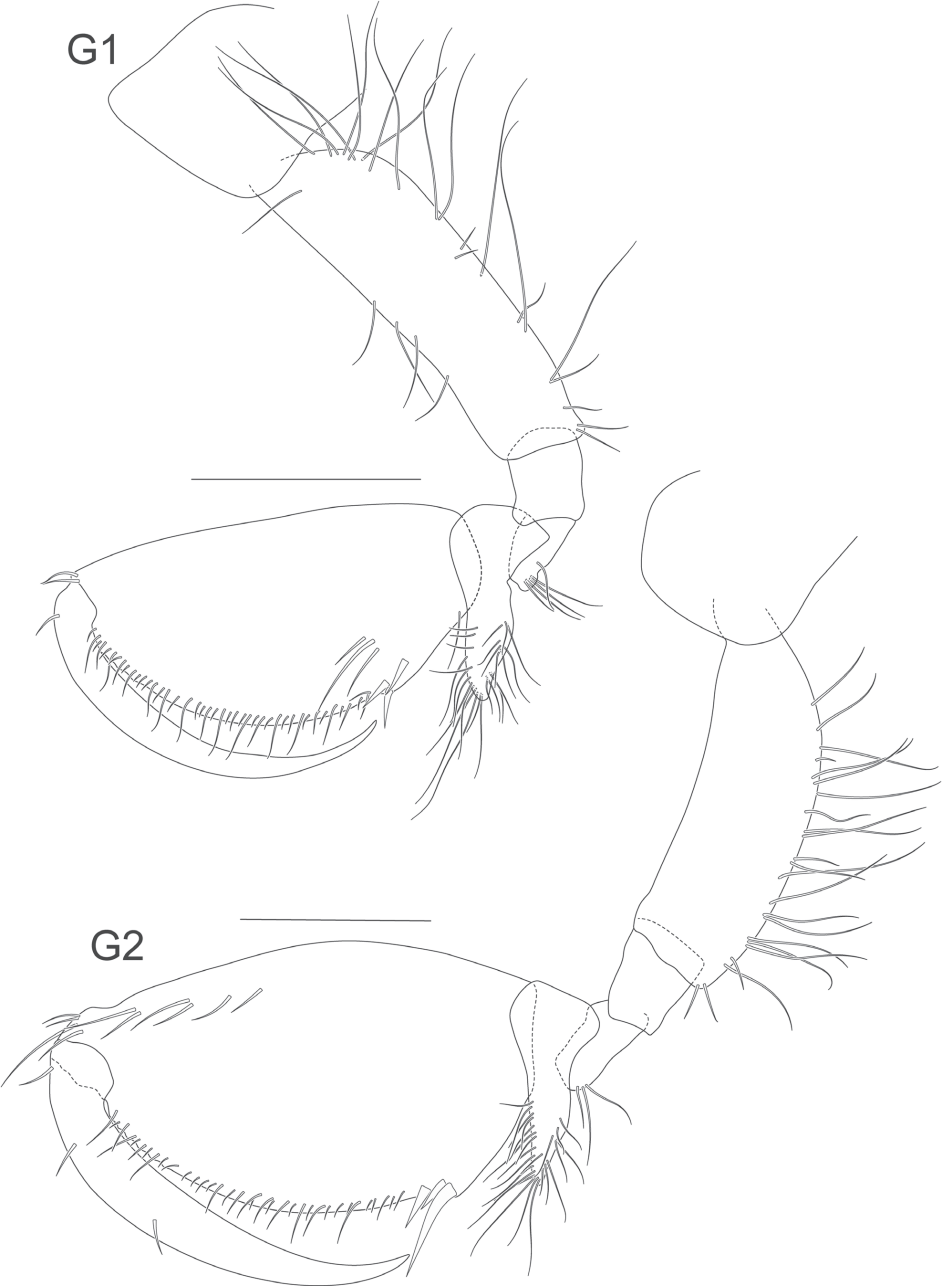
*Liljeborgia sibylline* sp. nov., holotype: SMF 62822, immature female, 6 mm, Clarion-Clipperton Zone. Scale bars: 0.5 mm (G1, G2).

**Figure 11.**
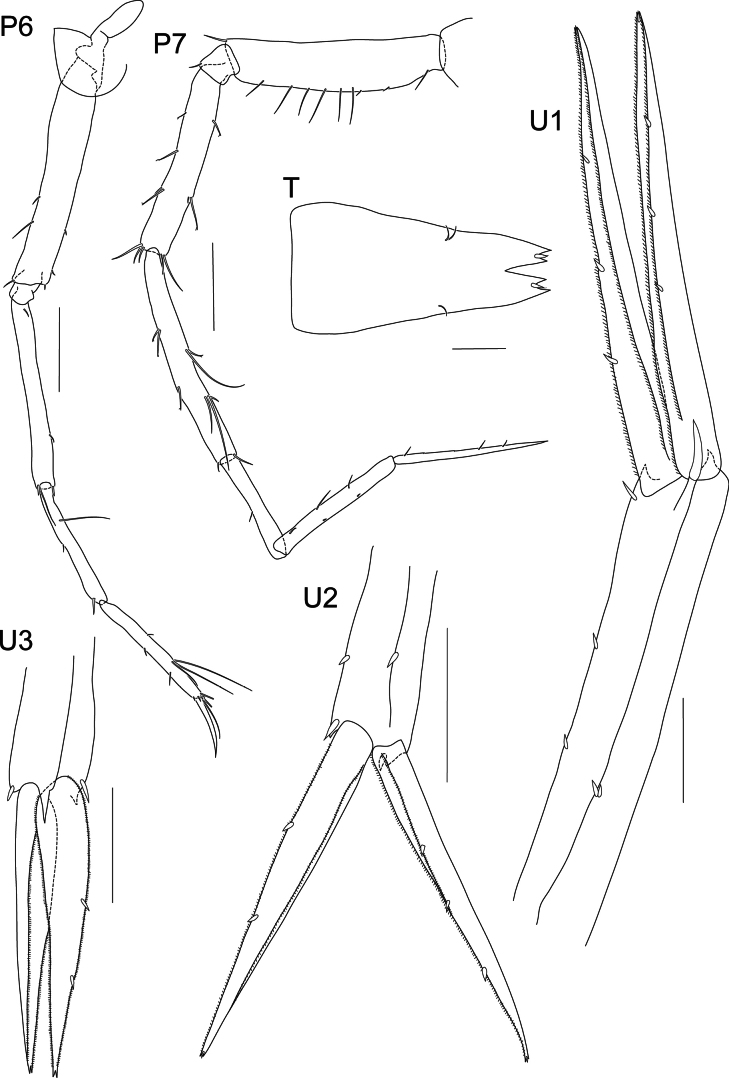
*Liljeborgia sibylline* sp. nov., holotype: SMF 62822, immature female, 6 mm, Clarion-Clipperton Zone. Scale bars: 0.5 mm (P6, P7); 0.1 mm (T); 0.2 mm (U1, U3); 0.3 mm (U2).

Figs 7, 8, 9, 10, 11

##### Type material.

***Holotype***: Pacific • Immature female, 6 mm, carcass and 2 slides, Clarion-Clipperton Zone, 11.819°N, 116.975°W; depth 4107 m; 29/04/2016; BGR contract area, “RV Kilo Moana”, MANGAN 2016, Station Ma 16-25, epibenthic sledge; SMF 62822; COI (PQ734720). ***Paratypes***: Pacific • Immature female (non-setose oostegites), 10.89 mm; Clarion-Clipperton Zone; 10.329°N, 117.197°W; depth 4283 m; 09/11/2020; NORI-D contract area, “RV Maersk Launcher”, Cruise 5A, Station STM_005, box core BC_349; NHMUK 2025.39 (Specimen 5658_TH_AMP1); COI (PV077099), 16S (PV077006), H3 (PV078010), 28S (PV077014). • Female (setose oostegites), 11.44 mm; Clarion-Clipperton Zone; 10.316°N, 117.514°W; depth 4396 m; 18/11/2020; NORI-D contract area, “RV Maersk Launcher”, Cruise 5A, Station SWM_024, box core BC_349; NHMUK 2025.40 (Specimen 5827_TH_AMP1).

##### Other material.

Pacific • Immature female, 6.63 mm; Clarion-Clipperton Zone; 10.325°N, 117.181°W; depth 4287 m; 30/11/2022; NORI-D contract area, “MV Island Pride”, Cruise 7B, Station TF_003, box core BC_476; DISCOLL-TMC-AMP-10187-a (Specimen 10187_TH_AMP1); COI (PV077100), 16S (PV077007), H3 (PV078011), 28S (PV077015). • Immature male, 6.53 mm; Clarion-Clipperton Zone; 10.845°N, 116.152°W; depth 4131 m; 04/11/2020; NORI-D contract area, “RV Maersk Launcher”, Cruise 5A, Station SPR_041, box core BC_337; DISCOLL-TMC-AMP-5267-a (Specimen 5267_TH_AMP1); COI (PV077098), 16S (PV077005), H3 (PV078009), 28S (PV077013).

##### Diagnosis.

Maxilliped palp article one with dorsal distal setae; article three anterior margin with setae in groups or pairs. Palmar margin of gnathopod 2 of young males lacking notch. Pereopod 3–4 propodus posterior border with long setae. Pereopods 5–7 bases very narrow, lacking crenulations on the posterior margin. Pleosomites 1–3 posterodorsal area produced into a tooth; epimera 1–3 with minute tooth in posterodistal corner. Uropod 1 peduncle dorsomedial margin with several spines. Telson very shallowly cleft, 18%, inter-teeth spine short with setae, barely reaching tip of medial tooth. Eyes absent.

##### Description.

Based on holotype, immature female, 6 mm. SMF 62822.

***Head*** (Figs 7, 8): ***Rostrum*** well-developed, reaching ~ 1/3 of A1 peduncle article 1. ***Eye*** absent, colourless in alcohol. ***Antenna 1*** primary flagellum with 12 (damaged in the holotype) to 22 articles (paratype NHMUK 2025.39); accessory flagellum with at least seven (damaged in the holotype) to 14 (paratype NHMUK 2025.39) articles. ***Antenna 2*** article four of peduncle with dorsomedial and ventrolateral spines and setae; article five with groups of setae on the dorsal margin and a few setae on the ventral margin, without spines; flagellum with ten articles.

***Mouthparts*** (Fig. 9): ***Lower lip*** covered in fine setules. ***Epistome & upper lip*** broadly rounded, prominent, extending beyond upper lip. ***Mandible***: left mandible incisor process nine-cuspidate, lacinia mobilis large, three-cuspidate; setal row of five spines; right mandible damaged, unknown; article one of palp length 4.5 × width (length article one: article two = 0.9); article two with three setae distomedially, length 6.5 × width; article three with three setae on the distomedial margin, one distolateral and three apically, length 4.08 × width, 0.68 × article two. ***Maxilla 1*** palp article two with two long setae on lateral margin, five slender spines on apical and medial margin, and three long facial setae; outer plate with nine spines, of which two are denticulate; inner plate lost. ***Maxilla 2*** setose, outer plate with two setae on lateral margin. ***Maxilliped*** inner plate with three apical spines and five marginal and submarginal setae (shown as setal bases); outer plate with seven normally-spaced short and stout spines on medial margin, interspersed with seven medioventral slender setae; palp article one without setae; palp article three 0.73 × article two; dactylus 0.87 × article three, with anterior and posterior margins distinctly curved, posterior margin covered with fine setules, anterior margin with setae in groups or pairs.

***Pereon*** (Figs 10, 11): ***Gnathopod 1*** coxa broadly quadrate; merus with five setae; carpus prolongation just reaching palmar defining spine group; propodus twice as long as wide; palm convex, with three palmar defining spines and small hooked spines and slender, long setae along margin; dactylus without teeth, with one seta proximally. ***Gnathopod 2*** coxa broadly rectangular; carpus prolongation not quite reaching palmar defining spine group; propodus length 1.8 × width; palm convex, with three palmar-defining spines, one longer than the other two, and small hooked spines and slender, long setae along margin; with a row of setae along the anterodistal margin; dactylus without teeth, with three setae along the length. Gnathopod 2 larger than gnathopod 1; gnathopod 2 propodus: gnathopod 1 propodus length = 1.4. ***Pereopod 3*** coxa with small posteroventral notch with one seta, one seta at anteroventral margin; basis posterior margin with seven setae; merus 1.44 × carpus, 1.06 × propodus; carpus length 6.5 × width; propodus length 8.8 × width; dactylus slender, slightly curved, medium length, 0.7 × carpus, 0.54 × propodus; propodus with long setae along posterior margin small spine distally. ***Pereopod 4*** coxa with anteroventral notch with one seta; basis with three setae along posterior margin; merus subequal to carpus and 0.94 × propodus; carpus length 5.4 × width; propodus length 8.9 × width; dactylus slender, slightly curved, medium length, 0.81 × carpus and 0.59 × propodus; propodus with long setae along posterior margin; anterior border with one small seta. ***Pereopod 5*** basis very narrow (length 7.0 × width), posterodorsal margin with five short setae; merus with one seta on the posterior margin and three setae distally. Distal articles missing. ***Pereopod 6*** basis very narrow (length 5.5 × width), with three short setae posteriorly and three short setae anteriorly; carpus 0.68 × merus; propodus with three very long setae posteriorly, dactylus slender, slightly curved, 0.47 × propodus. ***Pereopod 7*** basis very narrow (~ 4.4 × width), nine setae along posterior margin, one spine anterodistally; merus and carpus with long anterior and posterior setae, carpus 1.17 × merus; propodus 1.84 × pereopod 6 propodus, with short setae on posterior and anterior margins; dactylus long, straight, with five setae along posterior margin, 0.6 × propodus.

***Pleosome*** (Figs 7, 11): ***Pleonites 1*–*3*** carinate with small tooth mid-dorsally on posterior margin. ***Pleonite 1*** posterodorsal margin with small tooth; epimeron 1 with minute tooth (paratype NHMUK 2025.40), posterior border slightly convex. ***Pleonite 2*** posterodorsal area produced into a tooth; epimeron 2 with a minute posterodistal tooth, with posterior border slightly convex. ***Pleonite 3*** posterodorsal area produced into a large tooth; epimeron 3 rounded with minute tooth in the posterodistal corner, with posterior border almost straight (very weakly convex).

***Urosome*** (Figs 7, 11): ***Urosomite 1*** with distinct middorsal tooth. Uropod 1 peduncle with two dorsolateral spines and one spine distally; two dorsomedial spines, outer ramus with three well-developed lateral spines and no medial spines, one small spine at the tip of ramus; inner ramus without lateral spines and with three well-developed spines medially, one small spine at the tip of ramus; lateral and medial margins of both rami microsetose. ***Urosomite 2*** with distinct mediodorsal tooth. ***Uropod 2*** peduncle with two dorsolateral spines and two dorsomedial spines; outer ramus with two short, large spines, one small spine at the tip of ramus; inner ramus with three short spines on medial margin, one small spine at the tip of ramus; lateral and medial margins of both rami microsetose. ***Urosomite 3*** without mediodorsal tooth. ***Uropod 3*** peduncle damaged, with one medial and one lateral spine dorsally; outer ramus without spines; inner ramus with two stout spines on inner margin; lateral and medial margins of both rami microsetose. ***Telson*** cleft to 18% of its length; outer tooth of each lobe overreaching medial tooth; inter-teeth spine short with setae, barely reaching tip of medial tooth, two setae present on one lobe, and one on the other.

##### Sexual dimorphism.

Setose oostegites present on pereonites 2–5.

##### Etymology.

The species name *sibylline*, used as a noun in apposition, means puzzling, strange, peculiar, or curious. It is in honour of the usage of this word by Cedric d’Udekem d’Acoz in his 2008 *Liljeborgia* paper; a word that we were unfamiliar with, just as we were with this new species.

##### Remarks.

*Liljeborgia sibylline* sp. nov. belongs to the subgenus *Lilljeborgiella* (d’Udekem d’Acoz 2010a). The peduncle of uropod 1 has several spines along the dorsomedial margin. Groups of setae are present on the anterior margin of maxilliped palp article three. The posterior border of the propodi of pereopods 3 and 4 have long setae, and the bases of the pereopods 5–7 are narrow. Although the holotype specimen does not appear to possess the *Lilljeborgiella* characteristic distal outer setae on the first article of the maxilliped palp, there are four long setae present on article 1 of the mature female paratype NHMUK 2025.40. It is possible that the smaller holotype specimen is damaged or these setae were not visible in the slide preparation.

The new species is morphologically most similar to *Liljeborgia holthuisi*, as is *L. scylla* sp. nov. (Table 1). The main difference between these species is the extremely narrow basis of pereopod 7 in *L. sibylline* sp. nov. (length 4.4 × width in *L. sibylline* sp. nov. and 2.58 × in *L. holthuisi*). The pleonites 1–3 have a distinct mid-dorsal tooth in the new species, while the tooth in *L. holthuisi* is much smaller and followed by a rounded edge. *Liljeborgia sibylline* sp. nov. has a single larger distinct middorsal teeth on urosomites 1 and 2, while *L. holthuisi* has a middorsal carina with two very small teeth. The small protrusion on the gnathopod 2 of *L. holthuisi* is absent in *L. sibylline* sp. nov.

Both species were collected from the deep sea, *L. sibylline* sp. nov. was found at depths of 4107–4287 m, while *L. holthuisi* was collected at even greater depths of 5825–5841 m (d’Udekem d’Acoz, 2010b). Even though both species were collected at abyssal depths in the Pacific Ocean, their collection sites are separated by more than 4500 km.

Another species that shares characteristics with *L. sibylline* sp. nov. is *L. cota*. Its telson is cleft at 34% of its length, compared to the even smaller cleft telson of *L. sibylline* sp. nov. that is cleft only 18% of its length. Both species originate from the Pacific Ocean, but differ in their depth distribution, with *L. sibylline* sp. nov. found at 4107 m depth, while *L. cota* was collected at 366–2000 m (J.L. Barnard, 1962, 1967, 1971). The main difference between these two species is the posteroventral corner of pleonites 1–3, which is rounded in the new species, whereas *L. cota* bears a tooth. The bases of pereopods 5–7 are also more narrow in *L. sibylline* sp. nov.

##### Distribution.

Known only from the Clarion-Clipperton Zone, 4107–4396 m depth.

##### Molecular data.

Sequence data for the holotype of *Liljeborgia sibylline* sp. nov. is deposited in GenBank under accession number PQ734720. The species has also received a Barcode Index Number from Barcode of Life Data Systems: BOLD: AEA9975 (https://doi.org/10.5883/BOLD:AEA9975).

## Discussion

The two newly described *Liljeborgia* species from the Clarion-Clipperton Zone (CCZ) are morphologically very similar to each other. Neither species has teeth on the dactylus of gnathopods 1 and 2, and both have a very shallow cleft of the telson (less than 30%) and distinct teeth on pleonites 1–3. The posterior margin of the bases of pereopods 5–7 in both species lacks the crenulations commonly found in many *Liljeborgia* species, and the bases of these pereopods is overall extremely narrow.

The main morphological differences between the two species are the notch on palmar margin of the gnathopod 2 in young males (present in *L. scylla* sp. nov., but absent in *L. sybilline* sp. nov.); the posterodistal corner of the epimera 1–3 (*L. sibylline* sp. nov. is rounded with a minute tooth, while *L. scylla* sp. nov. lacks these minute teeth); and the basis of pereopod 7, which is slightly narrower in *L. sibylline* sp. nov. than in *L. scylla* sp. nov.

The overall morphological similarity between *L. scylla* sp. nov. and *L. sibylline* sp. nov. shows the importance of genetic data in differentiating morphologically cryptic species. Although *L. scylla* sp. nov and *L. sibylline* sp. nov. do not have striking morphological differences, there is a significant divergence in their COI sequences.

In the genus *Liljeborgia* there are very few species with a shallowly cleft telson (less than 30% of its length). *Liljeborgia scylla* sp. nov., *L. sibylline* sp. nov., *L. holthuisi*, and *L. cota* share this unusual characteristic, which easily separates them from all other species of *Liljeborgia*. There are a few species within the genus that have tooth-like protrusions on the palm of the gnathopod 2. *Liljeborgia scylla* sp. nov., *L. holthuisi*, *L. cota*, *L. quadridentata*, and *L. quinquedentata* have varying numbers of these tooth-like protrusions. This characteristic is observed in males, while the palm of females is smooth (Barnard 1962; d’Udekem d’Acoz 2008). In *L. cota* and *L. quinquedentata*, the protrusions have been observed to be larger in younger males than in older males, indicating that the protrusions might be bigger in juvenile males of all species with this characteristic. *Liljeborgia holthuisi* was described on the basis of a single adult male specimen, 10.5 mm in length, which had a very small protrusion on the palm border of gnathopod 2. This protrusion may therefore be bigger in juveniles of that species and could potentially resemble the gnathopod 2 of the young male of *L. scylla* sp. nov. illustrated here. Further study of individuals of varying sizes and sexes of these two species is needed to clarify the species-level differences in this character.

## Supplementary Material

XML Treatment for Liljeborgia (Lilljeborgiella) scylla

XML Treatment for Liljeborgia sibylline

## Additional information

### Author ORCIDs

Roxana Timm https://orcid.org/0000-0002-7751-6801

Eva C. D. Stewart https://orcid.org/0000-0001-8383-5705

Anne-Nina Lörz https://orcid.org/0000-0001-9942-349X

Tammy Horton https://orcid.org/0000-0003-4250-1068

### Data availability

All of the data that support the findings of this study are available in the main text.
